# Hyperbaric oxygen for the treatment of traumatic incomplete spinal cord injury: a systematic review and meta-analysis of randomized controlled trials

**DOI:** 10.4103/mgr.MEDGASRES-D-25-00250

**Published:** 2026-09-12

**Authors:** Jiantao Shi, Hongfei Ge, Zhouyang Jiang, Guangjian He, Liang Tan, Rong Hu, Shengli Hu

**Affiliations:** 1 Department of Neurosurgery and Key Laboratory of Neurotrauma, The First Affiliated Hospital (Southwest Hospital) of Army Medical University, Chongqing, China; 2 Center of Critical Care Medicine, The First Affiliated Hospital (Southwest Hospital) of Army Medical University, Chongqing, China

**Keywords:** activities of daily living, ASIA score, evidence-based medicine, hyperbaric oxygen therapy, meta-analysis, motor function, neurorehabilitation, sensory function, systematic review, traumatic incomplete spinal cord injury

## Abstract

The therapeutic efficacy of hyperbaric oxygen therapy for traumatic incomplete spinal cord injury remains a subject of debate. To comprehensively evaluate the impact of hyperbaric oxygen on motor function, sensory function, and activities of daily living in patients with traumatic incomplete spinal cord injury, we conducted a systematic review and meta-analysis. We performed a comprehensive search across PubMed, Embase, the Cochrane Library, Web of Science, and major Chinese databases for randomized controlled trials published through June 2025. A total of 15 randomized controlled trials were included. The pooled results indicated that hyperbaric oxygen therapy significantly improved the American Spinal Injury Association motor score (mean difference = 10.30, 95% confidence interval: 7.70–12.90), the total American Spinal Injury Association sensory score (mean difference = 29.29, 95% confidence interval: 18.82–39.75), the American Spinal Injury Association light touch score (mean difference = 9.84, 95% confidence interval: 6.15–13.53), and the American Spinal Injury Association pinprick score (mean difference = 8.75, 95% confidence interval: 4.40–13.11). Furthermore, hyperbaric oxygen therapy was associated with significant enhancements in the Barthel Index (mean difference = 16.60, 95% confidence interval: 10.51–22.68) and the Functional Independence Measure (mean difference = 17.41, 95% confidence interval: 10.83–23.98). In conclusion, adjunctive hyperbaric oxygen therapy appears to improve motor function, sensory function, and activities of daily living in patients with traumatic incomplete spinal cord injury compared to conventional treatments alone. However, due to methodological limitations and potential publication bias in the included studies, these findings should be interpreted with caution, and further high-quality, multi-center randomized controlled trials are warranted.

## Introduction

Traumatic spinal cord injury (SCI) refers to structural and functional impairment of the spinal cord induced by external forces.^1^ Complete SCI typically exhibits an extremely poor prognosis, primarily due to irreversible neural damage.^2^ For patients with incomplete SCI (iSCI), secondary injury progression is sometimes clinically manifested as neurological deterioration within the first 8–12 hours post-trauma.^3^,^4^ Spinal cord edema initiates within minutes after traumatic injury, reaches its peak at 3 to 5 days post-injury, and gradually subsides to baseline within 7 days.^5^ Therefore, timely and effective intervention targeting secondary injury is of great significance for improving the prognosis of patients with traumatic iSCI.

Ischemia-hypoxia represents the most critical mechanism underlying secondary SCI,^6^ therefore, theoretically, treatments that increase oxygen tension at the injured site may facilitate neurological recovery. Hyperbaric oxygen (HBO) therapy is a well-established intervention for hypoxic disorders, involving the administration of pure or high-concentration oxygen via a mask or within a hyperbaric chamber maintained at an ambient pressure exceeding 1.4 atmospheres absolute (ATA). Clinically, HBO therapy is currently widely applied for ischemia-hypoxia-related conditions and has demonstrated efficacy in managing carbon monoxide poisoning, necrotizing soft tissue infections, decompression sickness, gas gangrene, and craniocerebral trauma.^7^,^8^ In 1977, Holbach et al.^9^ were the first to observe that HBO therapy could improve postoperative neurological dysfunction in patients with traumatic SCI. Despite its long history of application in traumatic SCI, the therapeutic efficacy of HBO therapy for this condition remains controversial. Gamache et al.^10^ reported that HBO therapy failed to improve the final neurological outcome in patients with acute spinal cord injury. This study aimed to evaluate the effects of HBO therapy on motor function, sensory function, and activities of daily living in patients with traumatic SCI, which are the most clinically relevant outcomes for those with iSCI. We systematically identified and synthesized randomized controlled trials (RCTs) of patients with traumatic iSCI who received HBO therapy and conducted a meta-analysis to assess the efficacy of HBO therapy for traumatic iSCI.

## Methods

We performed a meta-analysis based on the Preferred Reporting Items for Systematic Reviews and Meta-Analyses (PRISMA) guidelines.^11^

## Search strategy

We searched studies published between January 2015 and June 2025 at PubMed, Embase, Cochrane Library, Web of Science, China National Knowledge Infrastructure, WanFang Data, and VIP databases, with the following terms: ((Hyperbaric Oxygen) OR (Hyperbaric Oxygen Therapy) OR (Hyperbaric Oxygen Therapies) OR (Hyperbaric Oxygenation) OR (HBO) OR (HBOT)) AND ((Spinal Cord Injury) OR (Spinal Cord Injuries) OR (Spinal Cord Trauma) OR (Traumatic incomplete Spinal Cord Injury) OR (incomplete Spinal Cord Injury) OR (Spinal Cord Transections) OR (Spinal Cord Laceration) OR (Spinal Cord Lacerations) OR (Spinal Cord Contusion) OR (Spinal Cord Contusions) OR (iSCI) OR (SCI)). Additionally, we reviewed the references of relevant studies to identify potential articles.

### Study selection

Studies were included in this review if they met the following criteria: (1) RCTs, with experimental (received HBO therapy in conjunction with conventional therapy) and control (received conventional therapy only) groups; (2) Participants had traumatic iSCI confirmed by computed tomography or magnetic resonance imaging, with a definite history of trauma; participants with non-traumatic SCI were excluded; (3) Interventions involved HBO therapy; and (4) The study reported at least one of the following clinical outcomes: American Spinal Injury Association (ASIA) motor score,^12^ ASIA sensory score,^13^ Barthel index (BI),^14^ modified Barthel index (MBI),^15^ or functional independence measure (FIM).^16^ Studies were excluded in this review if they met the following criteria: (1) participants had non-traumatic SCI from neoplasm, infection, inflammation, degenerative disease, congenital malformation, vascular lesions, or iatrogenic injury; (2) enrolled only patients with complete SCI, or did not report disaggregated data for the iSCI subgroup; (3) lacked sufficient quantitative outcome data, had incomplete follow-up, or provided no extractable data for synthesis; (4) duplicate publications, overlapping patient cohorts, or trial protocols without final outcome data; (5) unclear HBO intervention parameters, treatment course, or follow-up duration, or follow-up shorter than 4 weeks.

Two authors (JS and SH) screened the titles and abstracts of all studies after removing duplicates, excluding those that did not meet the criteria. The full texts of the remaining articles were then downloaded for further evaluation based on the inclusion criteria. In cases of disagreement, the researchers engaged in discussion to reach a consensus; a third author (HG) was consulted when necessary. Studies published in languages other than English were translated for review.

### Data extraction

Literature was imported into EndNote in batches for duplicate removal, followed by a thorough screening process. Two authors (JS and SH) independently extracted the following data: study design, first author, journal, year of publication, patient characteristics, surgical co-interventions and timing, injury severity, sample size, attrition rates in each group, follow-up duration, and details of both experimental and control interventions. Outcome measures included the ASIA motor scores, ASIA sensory scores, BI, MBI, and FIM. The full texts of the studies were subsequently reviewed to confirm eligibility, and relevant information was extracted using a predefined data extraction form.

### Bias risk assessment

Bias risk was independently evaluated by two authors (ZJ and LT) using the Cochrane’s Risk of Bias Tool 2.0 (RoB 2, Current Official Recommended Version; https://sites.google.com/site/riskofbiastool/welcome/rob-2-0-tool).^17^ This assessment included random sequence generation, allocation concealment, blinding of participants and personnel, blinding of outcome assessment, incomplete outcome data, selective reporting, and other bias. In cases of disagreement, the two researchers engaged in discussion to reach a consensus; if necessary, a third author (GH) was consulted to ensure a thorough and objective assessment.

### Outcome measures

The primary outcomes of this study were motor and sensory functions, assessed using the ASIA Motor Score, and ASIA Sensory Score. The ASIA Motor Score evaluates 11 representative muscles, yielding a maximum bilateral total score of 100. The ASIA Sensory Score encompasses both light touch and needling across 28 representative sensory areas, with a maximum bilateral total score of 224. Higher scores indicate better motor and sensory function.

Secondary outcomes focused on activities of daily living, measured using BI, MBI, and FIM. Key activities assessed included personal hygiene, bathing, feeding, toileting, stair climbing, dressing, bowel control, bladder control, ambulation, and chair-to-bed transfers.

### Statistical analysis

Statistical analysis was performed with RevMan 5.4 (Cochrane Collaboration, Oxford, UK). Data were calculated as the mean difference (MD) along with the corresponding 95% confidence intervals (CIs). A P-value of less than 0.05 was considered statistically significant. Heterogeneity was assessed using the *I*^2^ test; fixed-effect models were applied when heterogeneity was not significant (*I*^2^ < 50%), while random-effects models were utilized in cases of significant heterogeneity. Additionally, we followed PRISMA guidelines, Cochrane’s Risk of Bias Tool t was used to investigate potential publication bias. Sensitivity analyses were conducted using a one-by-one elimination method.

## Results

### Eligible studies and assessment of bias risk

A total of 783 studies were initially identified, from which 15 studies were ultimately included in the meta-analysis (**Figure 1**). Among these, 3 studies were published in English,^18^,^19^,^20^ while 12 were published in Chinese.^21^,^22^,^23^,^24^,^25^,^26^,^27^,^28^,^29^,^30^,^31^,^32^ The experimental group comprised 580 participants, whereas the control group included 578 participants. The results of the bias risk assessment are presented in **Figure 2**.

**Figure 1:**
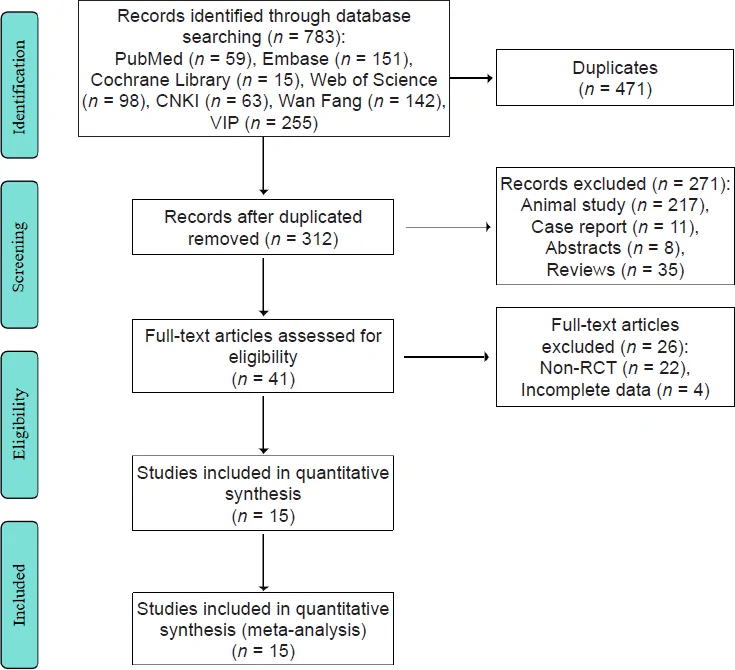
A flowchart of study screening. RCT: Randomized controlled trial.

**Figure 2:**
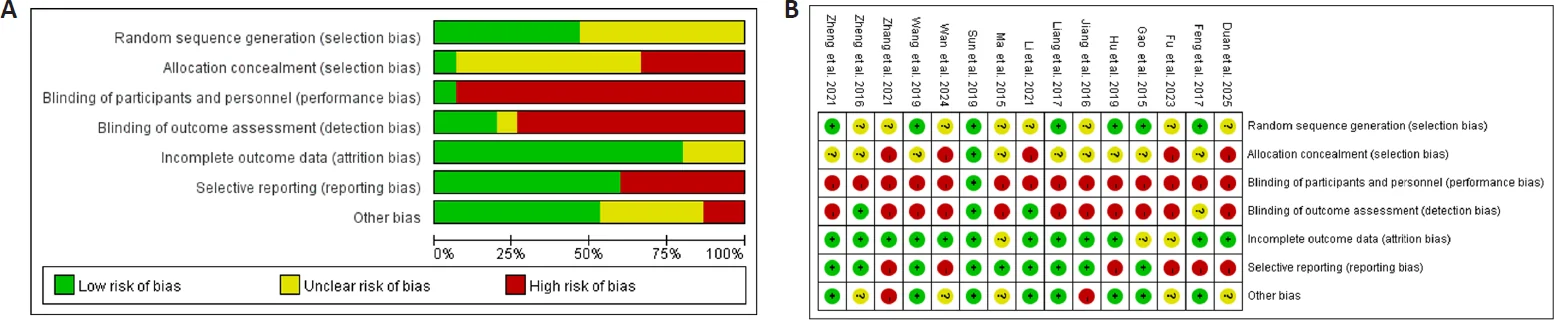
Risk of bias of the included studies. The risk of bias was assessed with RevMan 5.3. Each bias was reported as “low risk,” “high risk,” or “unclear risk” according to its quality of evidence. + indicates low risk of bias; ? indicates unclear risk of bias; – indicates high risk of bias.

### Study characteristics

The parameters for HBO therapy administered in the hospital are outlined in **Table 1** and **Additional Table 1**. A pressure of 0.2 MPa was utilized in 13 studies, while pressures of 0.24 and 0.25 MPa were employed in the other two studies. The duration of each HBO session ranged from 40 to 80 minutes, with treatment periods spanning 20 to 60 days.

**Table 1:** Characteristics of the included studies

Studies	Design	Participants (E/C)	Experimental group	Control group	Main outcomes	Other outcomes
Duan and Wang^21^	RCT	40/40	0.2 MPa, 80 min/session, daily, 30 sessions	Drugs, rehabilitation therapy	Barthel, FIM	RMT, MEP, SF-MPQ
Wan^22^	RCT	35/35	0.2 MPa, 60 min/session, daily, 10 times/course, 6 courses	Drugs, rehabilitation therapy, and surgical intervention	ASIA motor scale, ASIA sensory scale, Barthel	Effective rate
Fu et al.^23^	RCT	50/50	0.2 MPa, 60 min/session, daily, 10 sessions	Drugs, rehabilitation therapy, and surgical intervention	ASIA motor scale, ASIA sensory scale	SCIM, WHOQOL-BREF
Li et al.^18^	RCT	40/40	0.2 MPa, 60 min/session, daily, 30–40 sessions	Drugs, rehabilitation therapy	ASIA motor scale, ASIA sensory scale	IL-6, CRP, BDNF, BFGF
Zhang^24^	RCT	40/40	0.2 MPa, 60 min/session, daily, 20 sessions	Drugs, surgical intervention	MBI, FIM	Frankel scale
Zheng et al.^25^	RCT	30/29	0.2 MPa, 50 min/session, daily, 10 times/course, 6 courses	Drugs, rehabilitation therapy	ASIA motor scale, ASIA sensory scale, MBI	BDNF, side effect
Sun et al.^19^	RCT	41/38	0.2MPa, 60 min/session, daily, 30 days	Drugs, with (or without) surgical intervention	ASIA motor scale, ASIA sensory scale	HMGB1, NF-κB
Hu et al.^26^	RCT	42/42	0.24 MPa, 40 min/session, daily, 6 times/course, 8 courses	Drugs, rehabilitation therapy, and surgical intervention	ASIA motor scale, ASIA sensory scale	VAS, NGF, Neuritin
Wang et al.^27^	RCT	39/39	0.2 MPa, 60 min/session, daily, 6 times/course, 8 courses	Drugs, rehabilitation therapy, and surgical intervention	ASIA motor scale, ASIA sensory scale	Frankel, VAS, BDNF, NGF, TNF-α, CRP, PCT
Feng and Li^20^	RCT	20/20	0.2 MPa, 60 min/session, daily, 6 times/course, 8 courses	Drugs, rehabilitation therapy	ASIA motor scale, ASIA sensory scale, FIM	HAMD, HAMA
Liang et al.^28^	RCT	50/50	0.2 MPa, 60 min/session, daily, 10 times/course, 3 courses	Drugs, surgical intervention	ASIA motor scale, ASIA sensory scale	Side effect
Jiang et al.^29^	RCT	43/43	0.2 MPa, 60 min/session, daily, 10 times/course, 2 courses	Drugs, surgical intervention	ASIA motor scale, ASIA sensory scale	Frankel
Zheng and Zhang^30^	RCT	46/48	0.25 MPa, 80 min/session, daily, 10 times/course, 3 courses	Drugs, surgical intervention	ASIA motor scale, ASIA sensory scale	Effective rate
Gao et al.^31^	RCT	40/40	0.2 MPa, 80 min/session, daily, 30 sessions	Drugs, surgical intervention	ASIA motor scale, ASIA sensory scale	Effective rate
Ma^32^	RCT	24/24	0.2 MPa, 80 min/session, daily, 10 times/course, 3 courses	Drugs, rehabilitation therapy, and surgical intervention	ASIA motor scale, ASIA sensory scale, FIM	Effective rate

BDNF: Brain-derived neurotrophic factor; bFGF: basic fibroblast growth factor; C: Control group; CRP: C-reactive protein; E: experimental group; HAMD: Hamilton depression scale; HAMA: Hamilton anxiety scale; HMGB1: high mobility group box 1; IL-6: interleukin-6; MBI: modified Barthel index; MAS: modified Ashworth scale; MEP: motor evoked potential; NGF: nerve growth factor; NF-κB: nuclear factor kappa-B; PCT: procalcitonin; RMT: resting motor threshold; SCIM: spinal cord independence measure; SF-MPQ: short-form of McGill pain questionnaire; TNF-α: tumor necrosis factor-α; VAS: visual analog scale; WHOQOL-BREF: World Health Organization quality of life assessment scale

**Additional Table 1:** Characteristics of individual studies

Study	Sex (M/F)	Mean age (yr, E/C)	Damaged segment (E/C)	Time of initiation (hours post-injury)
Duan and Wang^21^	43/37	46.25±11.62/46.17±11.54	NR	NR
Wan^22^	NR	53.35±4.23/52.45±3.52	NR	NR
Fu et al.^23^	76/24	72.28±3.89/70.74±4.18	C 6, T 19, L 25/C 7, T 17, L 26	NR
Li et al.^18^	NR	NR	NR	NR
Zhang^24^	43/37	38.11±10.04/38.14±10.11	C 5, T 16, L 19/C 6, T 14, L 20	NR
Zheng et al.^25^	36/24	42±10/39±9	T 20, L and S 10/T 15, L and S 15	NR
Sun et al.^19^	NR	53.74 ± 11.82/51.46 ± 13.82	NR	NR
Hu et al.^26^	61/33	31.23 ±2.19/31.78 ±2.23	NR	NR
Wang et al.^27^	54/24	46. 75 ± 1.48/46. 42 ± 1. 57	T 27, L 12/T 25, L 14	NR
Feng and Li^20^	27/13	36.1±5.2/33.1±4.6	C 7, T 8, L 5/C 7, T 9, L 4	NR
Liang et al.^28^	57/43	43.5±8.9/40.0±9.1	C 15, T 21, L 14/C 15, T 22, L 13	NR
Jiang et al.^29^	50/42	36.42±5.17/36.13±5.08	C 5, T 16, L 22/C 6, T 14, L 23	NR
Zheng and Zhang^30^	59/35	36.4±12.3/37.8±13.1	NR	NR
Gao et al.^31^	52/28	42.6±8.8	C32, T18, L30	72
Ma^32^	25/23	42.1±2.0/43.2.1±2.6	C9, T and L 15/C8, T and L 15	NR

C: Cervical; E/C: experimental group/control group; L: lumbar; M/F: male/female; NR: not reported; S: sacral; T: thoracic.

Results of meta-analysis

#### ASIA motor score

In a total of 13 trials assessing motor function using the ASIA Motor Scale, a random-effects model was employed for analysis due to considerable heterogeneity among studies. The results indicated that HBO therapy significantly enhanced motor function (*MD* = 10.30, 95% CI: 7.70 to 12.90, *I*^2^ = 91%; **Figure 3A**). The funnel plot analysis revealed asymmetry, implying the potential presence of publication bias (**Figure 3B**). This sensitivity analysis suggests that the ASIA Motor Score results are robust, and that the results remain significant after excluding specific studies, despite high heterogeneity, suggesting that these studies all played an important role in the overall effect (**Additional Table 2**).

**Figure 3:**
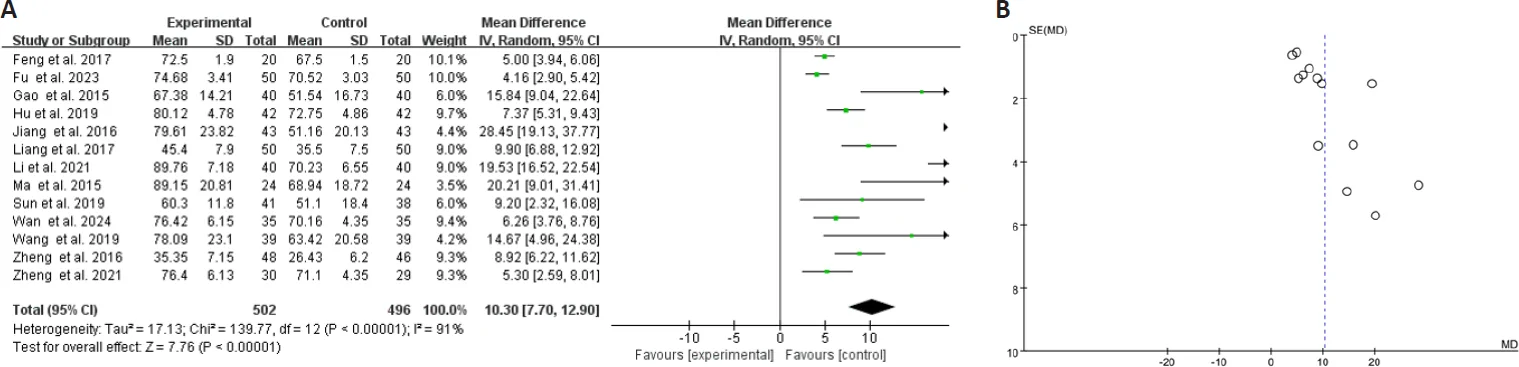
The meta-analysis of ASIA motor score in patients with traumatic iSCI. (A) Forest plot of ASIA motor score. HBO therapy combined with conventional treatments significantly improved the ASIA Motor Score in patients with traumatic iSCI. (B) Funnel plot of publication bias. ASIA: American Spinal Injury Association; HBO: hyperbaric oxygen; iSCI: incomplete spinal cord injury.

**Additional Table 2:** The sensitivity analysis of ASIA motor scores

Studies	Heterogeneity tests		Effect size consolidation	
	
	*P* value	*I* ^2^	MD (95% CI)	*P* value
Total studies	< 0.00001	91	10.30 (7.70, 12.90)	< 0.00001
Excluded Feng and Li^20^	< 0.00001	91	11.90 (8.03,14.35)	< 0.00001
Excluded Fu et al.^23^	< 0.00001	91	11.17 (8.17, 14.18)	< 0.00001
Excluded Gao et al.^31^	< 0.00001	92	9.91 (7.27, 12.56)	< 0.00001
Excluded Hu et al.^26^	< 0.00001	92	10.75 (7.84, 13.66)	< 0.00001
Excluded Jiang et al.^29^	< 0.00001	91	9.36 (6.88, 11.85)	< 0.00001
Excluded Liang et al.^28^	< 0.00001	92	10.38 (7.61, 13.15)	< 0.00001
Excluded Li et al.^18^	< 0.00001	83	8.56 (6.56, 10.56)	< 0.00001
Excluded Ma^32^	< 0.00001	92	9.91 (7.30, 12.53)	< 0.00001
Excluded Sun et al.^19^	< 0.00001	92	10.39 (7.69, 13.09)	< 0.00001
Excluded Wan^22^	< 0.00001	92	10.82 (7.96, 13.67)	< 0.00001
Excluded Wang et al.^27^	< 0.00001	92	10.10 (7.45, 12.75)	< 0.00001
Excluded Zheng and Zhang^30^	< 0.00001	92	10.51 (7.70, 13.32]	< 0.00001
Excluded Zheng et al.^25^	< 0.00001	92	10.89 (8.06, 13.72]	< 0.00001

ASIA: American Spinal Injury Association; CI: confidence interval; MD: mean difference.

#### ASIA sensory score

A total of seven RCTs reported the ASIA sensory score (total), while six trials reported the light touch and needling scores, respectively. Due to high heterogeneity, a random-effects model was employed for the analysis. The results indicated that HBO therapy significantly improved sensory function (*MD* = 29.29, 95% CI: 18.82 to 39.75, *I*^2^ = 95%; **Figure 4A**). For the six trials assessing light touch and needling, HBO therapy also significantly increased the light touch score (*MD* = 9.84, 95% CI: 6.15 to 13.53, *I*^2^ = 100%; **Figure 4B**) and the needling score (*MD* = 8.75, 95% CI: 4.40 to 13.11, *I*^2^ = 100%; **Figure 4C**). The funnel plot analysis of ASIA Sensory Scores exhibited asymmetry, implying the potential existence of publication bias (**Additional Figure 1**). Additionally, sensitivity analysis revealed that the systematic exclusion of individual studies did not significantly impact the pooled results for the total sensory score, the light touch score, and the needling score (**Additional Table 3**).

**Figure 4:**
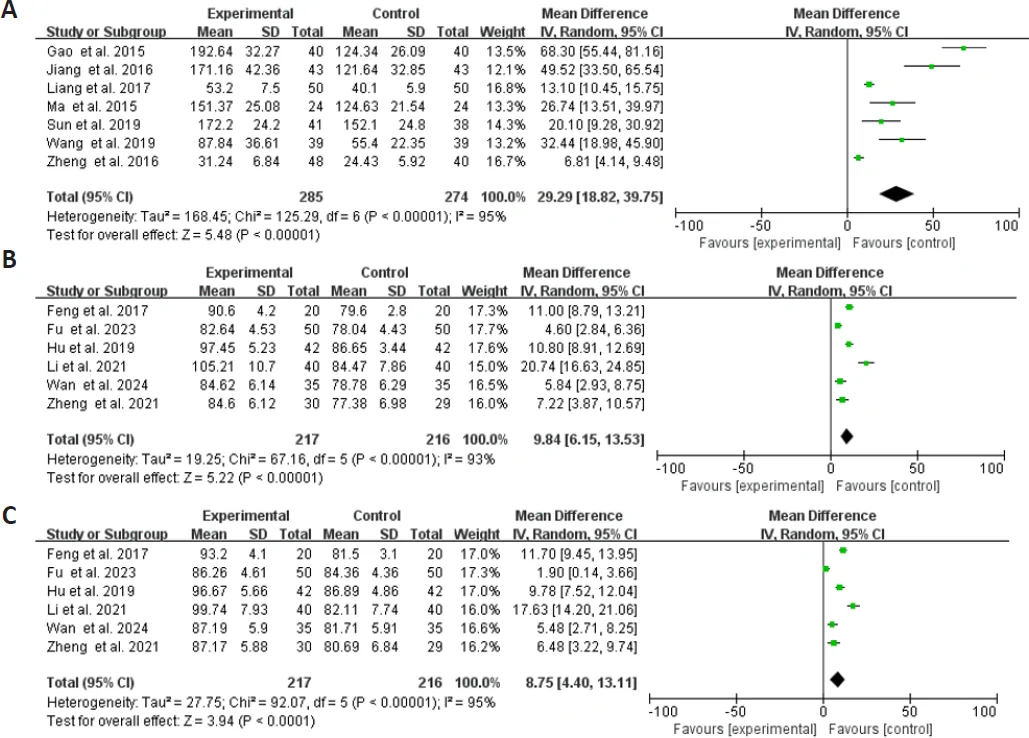
The meta-analysis of ASIA sensory score in patients with traumatic iSCI. (A–C) Forest plots of ASIA sensory score (A), ASIA sensory light touch score (B) and ASIA sensory needling score (C). HBO therapy combined with conventional treatments significantly increased the ASIA sensory, sensory light touch and sensory needling scores in patients with traumatic iSCI. ASIA: American Spinal Injury Association; HBO: hyperbaric oxygen; iSCI: incomplete spinal cord injury.

**Additional Table 3:** The sensitivity analysis of ASIA subscores

Studies	Heterogeneity tests		Effect size consolidation	
	
	*P* value	*I*^2^ (%)	MD (95% CI)	*P* value
**ASIA sensory score**				
Total studies	< 0.00001	95	29.29 (18.82, 39.75)	< 0.00001
Excluded Gao et al.^31^	< 0.00001	90	21.60 (13.79, 29.42)	< 0.00001
Excluded Jiang et al.^29^	< 0.00001	95	26.29 (15.83, 36.75)	< 0.00001
Excluded Liang et al.^28^	< 0.00001	96	33.54 (13.51, 53.56)	0.001
Excluded Ma^32^	< 0.00001	96	29.70 (18.39, 41.02)	< 0.00001
Excluded Sun et al.^19^	< 0.00001	96	30.93 (19.33, 42.53)	< 0.00001
Excluded Wang et al.^27^	< 0.00001	96	28.77 (17.64, 39.90)	< 0.00001
Excluded Zheng and Zhang^30^	< 0.00001	95	34.52 (17.22, 51.81)	< 0.00001
**ASIA light touch score**				
Total studies	< 0.00001	93	9.84 (6.15, 13.53)	< 0.00001
Excluded Feng and Li^20^	< 0.00001	94	9.64 (15.15, 14.12)	0.00001
Excluded Fu et al.^23^	< 0.00001	89	10.93 (7.21, 14.65)	< 0.00001
Excluded Hu et al.^26^	< 0.00001	93	9.68 (5.07, 14.30)	< 0.00001
Excluded Li et al.^18^	< 0.00001	88	7.93 (5.01, 10.85)	< 0.00001
Excluded Wan^22^	< 0.00001	94	10.65 (6.38, 14.92)	< 0.00001
Excluded Zheng et al.^25^	< 0.00001	94	10.36 (6.08, 14.64)	< 0.00001
**ASIA needling score**				
Total studies	< 0.00001	95	8.75 (4.40, 13.11)	< 0.00001
Excluded Feng and Li^20^	< 0.00001	95	8.16 (3.20, 13.12)	0.001
Excluded Fu et al.^23^	< 0.00001	89	10.17 (6.52, 13.81)	< 0.00001
Excluded Hu et al.^26^	< 0.00001	95	8.56 (3.23, 13.89)	0.002
Excluded Li et al.^18^	< 0.00001	93	7.06 (3.12, 11.00)	0.0004
Excluded Wan^22^	< 0.00001	96	9.42 (4.24, 14.60)	0.0004
Excluded Zheng et al.^25^	< 0.00001	96	9.21 (4.11, 14.30)	0.0004

ASIA: American Spinal Injury Association; CI: confidence interval; MD: mean difference.

#### BI, MBI, and FIM scores

A total of two trials reported the BI, two reported the MBI, and three reported the FIM. A random-effects model was selected for the analysis of BI, MBI, and FIM due to the absence of heterogeneity. However, HBO therapy was associated with a significant improvement in BI (*MD* = 16.60, 95% CI: 10.51 to 22.68, *I*^2^ = 100%; **Figure 5A**), MBI (*MD* = 16.93, 95% CI: 0.97 to 32.90, *I*^2^ = 100%; **Figure 5B**), and FIM (*MD* = 17.41, 95% CI: 10.83 to 23.98, *I*^2^ = 100%; **Figure 5C**). Funnel plot and sensitivity analysis were not conducted, as the limited number of included studies precluded valid detection of publication bias.

**Figure 5:**
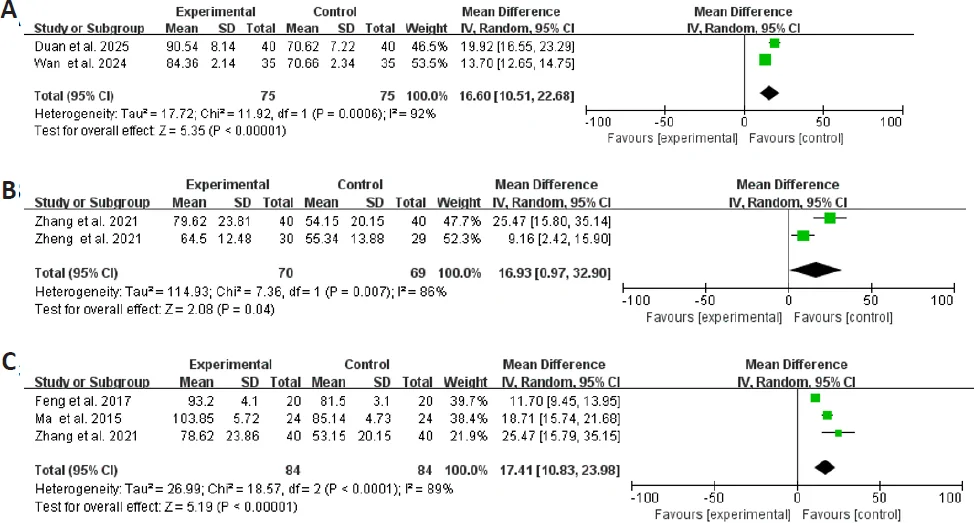
The meta-analysis of BI, MBI and FIM scores in patients with traumatic iSCI. (A–C) Forest plots of BI (A), MBI (B) and FIM (C). HBO therapy combined with conventional treatments significantly improved activities of daily living in patients with traumatic iSCI. ASIA: American Spinal Injury Association; BI: barthel index; FIM: functional independence measure; HBO: hyperbaric oxygen; mBI: modified barthel index; iSCI: incomplete spinal cord injury.

#### Subgroup analysis of ASIA motor scores

Subgroup analysis stratified by baseline conventional therapy revealed that HBO significantly improved ASIA motor scores both when added to rehabilitation plus pharmacotherapy (8 studies: *MD* = 8.84, 95% CI: 5.76 to 11.91, *I*^2^ = 93%) and when added to pharmacotherapy alone (5 studies: *MD* = 13.00, 95% CI: 8.42 to 17.58, *I*^2^ = 78%; **Figure 6**). The test for subgroup differences was not statistically significant (*P* = 0.14), indicating that the adjunctive efficacy of HBO is independent of concomitant rehabilitation interventions.

**Figure 6:**
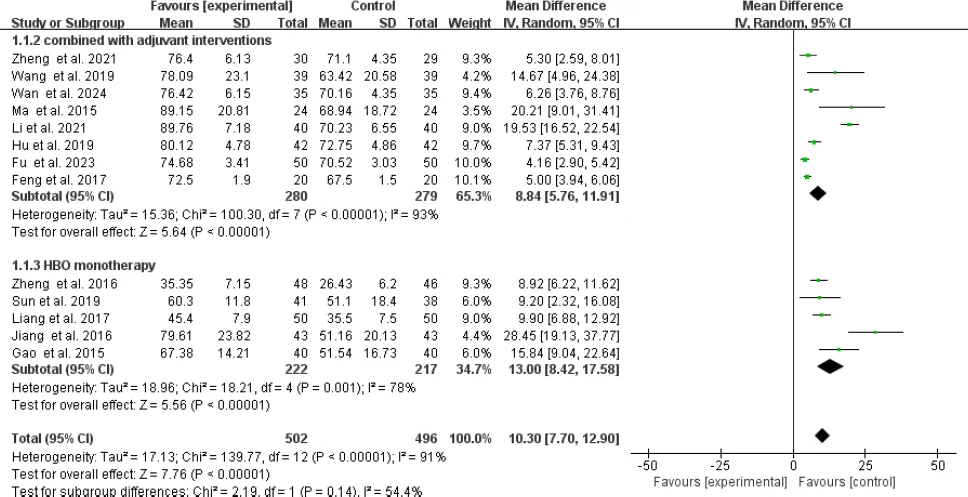
The subgroup meta-analysis of ASIA motor scores. The subgroup forest plot of ASIA motor scores revealed no significant between-subgroup difference (*P* = 0.14), indicating that the adjunctive effect of HBO is independent of concomitant rehabilitation. ASIA: American Spinal Injury Association; HBO: hyperbaric oxygen.

#### Safety and adverse events outcomes

This study rigorously extracted all endpoint data concerning the tolerance and safety of HBO therapy. Notably, no HBO-related fatalities were reported across any of the 15 trials included. However, due to the pervasive lack of safety reporting in the original studies, only one investigation^25^ performed a systematic evaluation.

## Discussion

To evaluate the role of HBO therapy in the treatment of traumatic iSCI, we conducted a systematic review of RCTs. A total of 15 high-quality RCTs involving 1158 traumatic iSCI patients were included, with the aim of clarifying the efficacy of HBO therapy in patients with traumatic iSCI. Our findings indicated that, compared with conventional treatments (e.g., standalone rehabilitation training and pharmacotherapy), HBO therapy combined with conventional treatments significantly improved the ASIA motor score, ASIA sensory score, BI, MBI, and FIM score in patients with traumatic iSCI. The characteristics observed in the forest plot, where all included studies reported positive outcomes, combined with the funnel plot suggested the presence of publication bias. This implies that studies yielding negative results may have been excluded, potentially leading to an overestimation of the current pooled effect sizes (ASIA motor score, *MD* = 10.30; ASIA sensory score, *MD* = 29.29). Additionally, the small sample size is a significant contributor to publication bias.

The conclusion of this study regarding the improvement of motor function in patients with traumatic iSCI by HBO therapy is consistent with the findings of a meta-analysis published by Huang et al.^33^ in 2021, which included studies up to May 2020. Huang’s meta-analysis also demonstrated that HBO therapy significantly increased the ASIA motor score (*MD* = 10.30, 95% CI: 7.70 to 12.90), ASIA sensory score (*MD* = 29.29, 95% CI: 18.82 to 39.75), and MBI (*MD* = 16.93, 95% CI: 0.97 to 32.90). However, our study further incorporated 6 high-quality RCTs published between 2020 and 2025 (involving a total of 469 patients) and excluded patients with non-traumatic SCI, focusing specifically on the effect of HBO therapy on neurological function in the subgroup of patients with traumatic SCI. Our study confirms that HBO therapy can improve the activities of daily living in patients with traumatic iSCI, thereby providing new intervention evidence to support the reduction of long-term disability risk in patients with traumatic iSCI.

Numerous experimental studies have demonstrated that HBO therapy exerts robust neuroprotective effects through a broad spectrum of physiological mechanisms, including the following: (1) Attenuation of cell death (apoptosis and ferroptosis): HBO therapy mitigates neuronal apoptosis by modulating apoptotic signaling pathways, including the brain-derived neurotrophic factor/tyrosine kinase receptor B pathway,^34^ caspase inhibition,^35^ and the suppression of matrix metalloproteinases-2/9.^36^ Additionally, HBOT inhibits ferroptosis by upregulating the solute carrier family 7 member 11 (SLC7A11)/glutathione peroxidase 4 pathway.^37^ (2) Antioxidant effects: HBOT effectively scavenges reactive oxygen species and restores redox balance through the regulation of superoxide dismutase/malondialdehyde.^38^ Additionally, it modulates the heme oxygenase-1/heat shock protein 32 pathway^39^ and activates the nuclear factor erythroid 2-related factor 2 signaling pathway.^40^ (3) Attenuation of neuroinflammation: HBOT mitigates excessive neuroinflammation by modulating multiple signaling pathways and influencing immune cell polarization, including the nuclear factor kappa-B/high mobility group protein box-1 pathway,^41^ Toll-like receptor 2/nuclear factor kappa-B pathway,^42^ macrophage polarization,^43^ and the NACHT-, LRR-, and PYD-containing protein 3 (NALP3) inflammasome.^44^ (4) Attenuation of spinal cord edema: it involves the downregulation of aquaporin 4 expression and the modulation of MMPs, which ultimately contributes to the reduction of spinal cord edema.^45^ (5) Promotion of angiogenesis: This is closely linked to the regulation of vascular endothelial growth factor.^46^ (6) Enhancement of autophagic degradation and clearance of injured or dysfunctional neurons.^47^ Collectively, these mechanisms support the notion that earlier initiation of HBO therapy is associated with more favorable recovery outcomes in patients with traumatic iSCI.

Our study has several limitations. First, although robust efficacy was confirmed using the standardized 0.2 MPa (2.0 ATA) protocol, substantial heterogeneity in treatment duration (10–60 sessions) precluded temporal dose–response meta-regression. Second, while subgroup analysis suggested that HBO remains effective as a standalone adjunct irrespective of concurrent rehabilitation, the variability in the composition of these interventions introduces confounding, underscoring the need for standardized control in future trials. Furthermore, allocation concealment was inadequately reported in two studies,^20^,^21^ increasing the risk of selection bias. Most included studies also lacked blinding of participants and personnel, contributing to potential performance bias. In several trials (e.g., Feng et al.^20^), outcome assessors were not blinded, raising the possibility of detection bias. The convergence of these methodological limitations may compromise the validity and generalizability of our findings, and the estimated effect of HBO on traumatic iSCI may be overestimated, warranting cautious interpretation. Furthermore, a glaring deficiency identified in the current literature is the pervasive underreporting of adverse events. With only one trial in our cohort actively monitoring safety endpoints, the seemingly benign profile of HBO must be interpreted with clinical caution. This underscores an urgent mandate for implementing PRISMA harms reporting standards in all subsequent SCI trials to establish a reliable risk-benefit ratio. Lastly, the pervasive reliance on aggregate continuous scores (such as the total ASIA motor scale) rather than tracking transition rates across distinct initial ASIA impairment categorical grades (e.g., from Grade C to D) severely obfuscates precision medicine efforts. Future multicenter RCTs must prioritize disaggregated, grade-specific reporting frameworks alongside the meticulous reporting of granular data on anatomical injury segments and precise intervention timing.

This systematic review and meta-analysis demonstrates that HBO therapy combined with conventional treatment can significantly improve neurological function and activities of daily living in patients with traumatic iSCI, compared with conventional treatment alone. However, the results should be interpreted with caution due to publication bias and methodological limitations of the included studies, especially the insufficient reporting of adverse events. High-quality, large-sample RCTs with standardized protocols are needed to further confirm the long-term efficacy and safety of HBO therapy for traumatic iSCI.

## Additional files:

***Additional Figure 1:*** *Funnel plot of publication bias for ASIA sensory scores.*

Additional Figure 1 Funnel plot of publication bias for ASIA sensory scores
(A–C) Funnel plots for total ASIA sensory score (A), ASIA sensory light touch score (B), and ASIA sensory needling score (C). Asymmetry in the plots suggests potential publication bias, which may be attributed to small sample sizes of included studies. ASIA: American Spinal Injury Association.

***Additional Table 1:*** *Characteristics of individual studies.*

***Additional Table 2:*** *The sensitivity analysis of ASIA motor score.*

***Additional Table 3:*** *The sensitivity analysis of ASIA subscores.*

**Facts**

1. Hyperbaric oxygen (HBO) alleviates hypoxia, attenuates oxidative stress and post-traumatic edema, and reduces ischemia–reperfusion injury in the spinal cord.
2. Adjuvant HBO therapy improves neurological recovery and daily function in patients with traumatic spinal cord injury, and early administration within an appropriate time window enhances its therapeutic effect.
3. HBO is well-tolerated and safe under standardized clinical protocols, supporting its utility as a supplementary rehabilitation intervention.

**Open Questions**

1. The optimal pressure, dosage, timing, and treatment course of HBO for spinal cord injury remain to be validated in large-scale randomized controlled trials.
2. It is unclear whether HBO synergizes with other neuroprotective or rehabilitative strategies to further improve long-term functional outcomes.
3. The exact molecular mechanisms underlying HBO-mediated neural protection and repair in spinal cord injury require further investigation.

## Data Availability

*All relevant data are within the paper and its additional files.*
